# Correspondence to the supplementary opinions on alternative cooling technologies in hot climate

**DOI:** 10.1007/s00484-018-1600-9

**Published:** 2018-08-27

**Authors:** Karin Lundgren-Kownacki, Elisabeth Dalholm Hornyanszky, Johanna Alkan Olsson, Per Becker

**Affiliations:** 1 Department of Design Sciences, Lund University, 221 00 Lund, Sweden; 2 Centre for Environment and Climate Research, Lund University, Lund, Sweden; 3 Division of Risk Management and Societal Safety, Lund University, Lund, Sweden

## Abstract

At present, air conditioning (AC) is the most effective means for the cooling of indoor space. However, its increased global use is problematic for various reasons. This is a correspondence to the supplementary opinion provided by Dr. Bin Yang, Dr. Stefano Schiavon, and Dr. Faming Wang to our paper titled “Challenges of using air conditioning in an increasingly hot climate.” The paper explored the challenges linked to increased AC use and discusses more sustainable alternatives. The supplementary opinion provides a great technical complement to our paper. However, there is a need for a more holistic view both when it comes to combining various solutions and involving various levels in society.

Our paper explores the societal challenges linked to increased air conditioning use and the problems associated with relying on one technology for cooling and discusses more sustainable alternatives and the societal responsibilities for implementing these. The supplementary opinion provided by Dr. Bin Yang, Dr. Stefano Schiavon and Dr. Faming Wang is a great technical complement to our paper by adding more energy-efficient solutions to our analysis of alternatives to air conditioning for cooling. The opinion gives more technical details and insights into how individuals can achieve thermal comfort in an energy efficient way. However, the aim of our paper was not to conduct a comprehensive assessment of cooling technologies, which we also state in the paper. The aim was to discuss challenges and potential alternatives using a transdisciplinary approach. Moreover, the analytical framework developed discusses alternative solutions and responsibilities at four societal levels—the individual, community, city, and national level. We argue that it is problematic to leave the responsibility to come up with cooling solutions entirely to the individual. We need a combination of solutions that are holistic and involve various levels in society.

